# Health of Criminal Women

**Published:** 1883-03-20

**Authors:** 


**Health of Criminal Women.**—Dr. E. M. Mosher, in an article upon this subject (Boston Medical and Surgical Journal), comes to the following conclusions:

1st. Intemperance and unchastity are the two vices which fill our penal institutions with women.

2d. The influence of these vices is detrimental to the health of the body, increasing its susceptibility to disease, and lessening its recuperative power.

3d. The diseases which follow as a direct result of these vices are syphilis, alcoholism, dyspepsia, rheumatism, and general anæmia.

4th. Morbid conditions of body react upon the moral nature, increasing and perpetuating the tendency to criminality; hence the importance of careful medical supervision as a reformatory measure.

5th. More ample provisions should be made in all large cities for the isolation and thorough treatment of venereal patients of *both sexes*, either by the addition of special wards to the general hospitals or by the establishment of hospitals for this class.

6th. The women who commit high crimes, that is, larceny, burglary, arson, manslaughter, etc., possess a more sensitive nervous organization than those who commit only offenses against chastity and public order.—*N. Y. Med. Record.*

